# Efficacy of dexamethasone intravitreal implant combined with anti-VEGF drugs in the treatment of diabetic macular edema: a systematic review and meta-analysis

**DOI:** 10.3389/fendo.2025.1673371

**Published:** 2026-01-05

**Authors:** Fang Chen, Xiaoqin Wang, Ming Chen, Mei Xin, Yiping Xian

**Affiliations:** Department of Ophthalmology, Chengdu First People’s Hospital/Chengdu Integrated TCM & Western Medicine Hospital, Chengdu, Sichuan, China

**Keywords:** diabetic macular edema, meta-analysis, anti-vascular endothelial growth factor, dexamethasone, best corrected visual acuity, central macular thickness

## Abstract

**Background:**

Diabetic macular edema (DME) remains a leading cause of vision loss in diabetic patients. Although anti-vascular endothelial growth factor (anti-VEGF) agents are first-line treatments, corticosteroids like dexamethasone (DEX) intravitreal implant have shown potential synergistic effects. This meta-analysis aimed to evaluate the efficacy and safety of DEX intravitreal implant combined with anti-VEGF agents compared to anti-VEGF monotherapy.

**Methods:**

A systematic search of PubMed, Embase, Web of Science, Science Direct, Wiley online, and Google scholar was performed up to April 2025. Primary outcomes were changes in best-corrected visual acuity (BCVA) and central macular thickness (CMT). Meta-analyses were performed using Review Manager (RevMan) version 4.4.1.

**Results:**

Eight studies comprising a total of 597 eyes were included in this meta-analysis. The pooled analysis demonstrated no statistically significant difference in BCVA between combination therapy and anti-VEGF monotherapy (Mean Difference [MD] = 1.79; 95% Confidence Interval [CI]: -1.68 to 5.26, *P* = 0.311). However, combination therapy resulted in a significantly greater reduction in CMT compared to anti-VEGF treatment alone (MD = -64.11 μm, 95% CI: -99.69 to -28.53, *P* < 0.001). The overall risk of bias across studies was rated as low to moderate. However, the incidence of adverse events is significantly higher in the combination therapy group.

**Conclusions:**

DEX combined with anti-VEGF agents confers superior anatomical outcomes in reducing CMT compared to anti-VEGF monotherapy in the management of DME. However, further large-scale, multicenter randomized controlled trials with extended follow-up are warranted to validate these findings and optimize treatment protocols.

## Introduction

Diabetic macular edema (DME) is one of the leading causes of visual impairment among individuals with diabetes mellitus (1). As the prevalence of diabetes continues to rise, the burden of DME on public health systems is expected to increase substantially (2). The pathogenesis of DME is multi-factorial, involving chronic hyperglycemia induced vascular dysfunction, breakdown of the blood-retinal barrier, and subsequent accumulation of fluid in the macula (3). Currently, intravitreal injection of anti-vascular endothelial growth factor (anti-VEGF) agents, such as ranibizumab, aflibercept, and bevacizumab, is the standard first-line treatment for DME. These agents have demonstrated significant efficacy in reducing macular edema and improving visual acuity. However, despite repeated anti-VEGF injections, a considerable proportion of patients exhibit incomplete responses or develop treatment resistance over time. In addition, the need for frequent injections imposes a significant burden on both patients and healthcare providers (4). In such cases of suboptimal response, switching from anti-VEGF therapy to an alternative treatment, such as the intravitreal dexamethasone (DEX) implant, represents a valuable clinical strategy (5). DEX intravitreal implant offer an alternative therapeutic strategy by targeting the inflammatory component of DME. DEX exerts its effects by down-regulating inflammatory cytokines, reducing vascular permeability, and stabilizing the blood-retinal barrier (6). Given the distinct yet complementary mechanisms of anti-VEGF agents and corticosteroids, combination therapy has been proposed as a means to enhance treatment efficacy, achieve better anatomical and functional outcomes, and potentially reduce the injection burden (7).

Several clinical studies have explored the efficacy and safety of combining DEX with anti-VEGF agents in the management of DME, yet their results remain heterogeneous and inconclusive (8). Some trials reported superior visual and anatomical improvements with combination therapy, while others found no significant advantage over anti-VEGF monotherapy (9). Therefore, a comprehensive meta-analysis of available evidence is warranted to systematically evaluate the therapeutic benefits and safety profile of DEX combined with anti-VEGF agents in DME treatment. This meta-analysis aims to synthesize current data to provide a clearer understanding of the role of combination therapy and investigates whether combination therapy yields superior outcomes compared to anti-VEGF monotherapy to guide future clinical practice.

## Methods

### Search strategy

This meta-analysis was conducted in accordance with the Preferred Reporting Items for Systematic Reviews and Meta-Analyses (PRISMA) guidelines. A systematic search of PubMed, Embase, Web of Science, Science Direct, Wiley online, and Google scholar was performed from database inception to April 2025. Search terms included combinations of “diabetic macular edema”, “DME”, “dexamethasone”, “anti-VEGF,” “bevacizumab”, “ranibizumab”, and “aflibercept”. Reference lists of relevant articles was manually screened to identify additional eligible studies.

### Inclusion and exclusion criteria

Studies were considered eligible if they met the following criteria:(1) research types:the literature selected encompassed only clinical controlled studies (RCTs) and quasi-randomized controlled studies (quasi-RCTs). (2) research subjects: patients diagnosed with DME. (3) grouping: The intervention group was treated with DEX combined anti-VEGF (e.g., bevacizumab, ranibizumab, or aflibercept), while the control group was only treated with anti-VEGF. (4) outcome measures: the primary outcome measures were the best corrected visual acuity (BCVA) and central macular thickness (CMT). Studies reporting at least one of the following outcomes: changes in BCVA in Early Treatment Diabetic Retinopathy Study (ETDRS) letters or logarithmic minimum angle of resolution (logMAR) values, CMT, intraocular pressure (IOP) from baseline, and ocular adverse events.

The exclusion criteria of the study papers were as follows: (1) macular edema of non-diabetic etiology. (2) studies not utilizing combined DEX and anti-VEGF therapy. (3)non-comparative studies, observational cohort studies, case-control studies, abstract articles, conference articles, and systematic reviews and meta-analyses. (4) studies with too small sample size less than 10 in each group.

### Data extraction

Two independent reviewers screened titles and abstracts, retrieved full-text articles, and extracted data using a standardized data extraction form. Extracted data included: study characteristics (authors, year of publication, study design, research location), patient demographics (age, sex, and sample size of DME patients, the count of eyes undergoing treatment), intervention details (drug types, follow-up duration), primary and secondary outcomes (BCVA, CMT, and IOP). The primary outcomes were changes in BCVA and CMT from baseline, while secondary outcomes were changes in IOP from baseline and the occurrence of adverse events. Given the heterogeneity in BCVA reporting, with some studies using ETDRS letters and others employing logMAR visual acuity, all data were harmonized into ETDRS letters for a valid analysis. The formula (ETDRS letters = 100 - (logMAR/0.02)) was used for conversion (10).

### Quality assessment

The risk of bias in the included studies was assessed using the Cochrane Risk of Bias Tool (ROB 2.0). Each domain was judged as “low risk,” “some concerns,” or “high risk.” Heterogeneity was assessed using the I² statistic and Cochran’s Q test, with I² > 50% indicating substantial heterogeneity. Sensitivity analyses were performed by sequentially excluding each study to evaluate the robustness of the results. Publication bias was assessed through visual inspection of funnel plots and quantified using Egger’s test (statistical significance set at p < 0.05).

### Statistical analysis

All meta-analyses were conducted using Review Manager (RevMan) version 4.4.1. Continuous outcomes were expressed as mean differences (MD) with 95% confidence intervals (CI). The choice between fixed-effects and random-effects models was determined based on the degree of heterogeneity. Specifically, the Mantel-Haenszel fixed-effects model was employed when I² ≤ 50%, indicating acceptable heterogeneity. In cases where I² > 50%, suggesting substantial heterogeneity, the Dersimonian-Laird random-effects model was applied to account for both within-study and between-study variability. This approach ensures methodological consistency while acknowledging potential clinical and methodological diversity across studies.

## Results

### Study selection and characteristics

**Figure 1:**
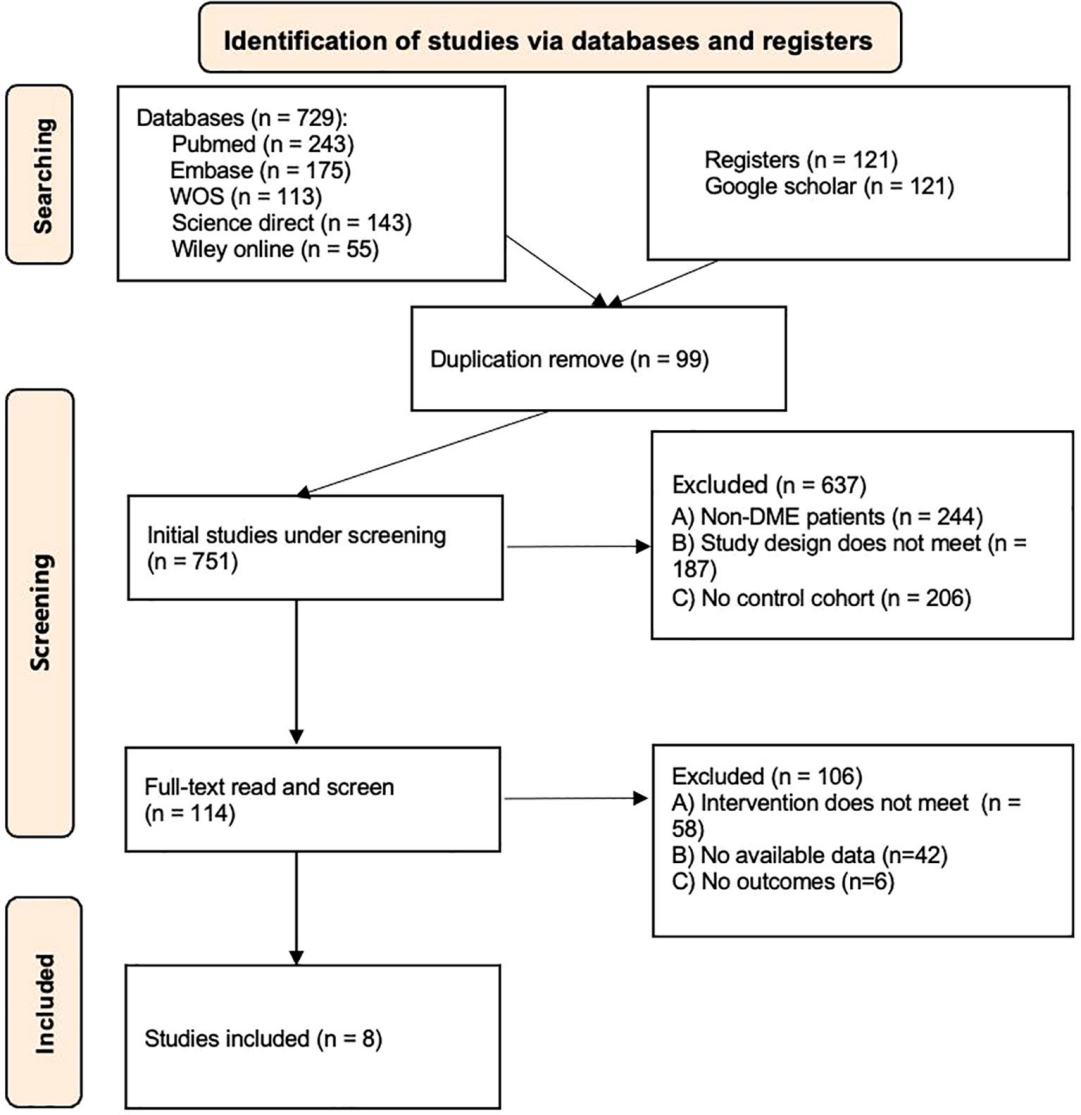
Flow diagram of the process of study selection.

The flow chart of study selection process is shown in **Figure 1**. Initially, 850 articles were retrieved (729 from online databases and 121 from Google Scholar). After deduplication and screening, eight articles were finally included for quantitative analysis (11–18). The basic characteristics, intervention measures and outcome indicators of all the literatures are listed in **Table 1**. A total of 525 patients (597 eyes) were included, among which 301 eyes were in the combination therapy group and 296 eyes were in the monotherapy group. Among the eight studies, there were four randomized controlled trial (RCT) studies and four quasi-RCT studies. The publication time ranged from 2018 to 2024. Regarding patient populations, six studies included individuals with treatment-naïve or general DME, while two studies focused specifically on persistent or refractory DME. In terms of intervention protocols, one study was treated with aflibercept combined with DEX, three studies with bevacizumab combined with DEX, and four studies with ranibizumab combined with DEX.

**Table 1:** Characteristics of included studies.

Study	Design	Location	Disease	Sample size (No.of people/eyes)	Treatment	Age (years)	Sample size (No.of people/eyes)	Follow-up Duration	Outcomes
Lin TC 2022 (11)	Quasi-RCT	China	DME	81/102	Aflibercept + DEX	65.1±8.7	41/52	6 months	①②③
					Aflibercept	63.4±13.9	40/50		
Limon U 2021 (12)	RCT	Turkey	Persistent DME	59/65	Bevacizumab + DEX	64.34 ± 8.7	29/35	6 months	①②
					Bevacizumab	63.20 ± 6.4	30/30		
Kaya M 2021 (13)	RCT	Turkey	DME	46/68	Ranibizumab+ DEX	64.6 ± 10.5	24/34	12 months	①②③④
					Ranibizumab	66.2 ± 8.8	22/34		
Maturi RK 2015 (14)	RCT	USA	DME	30/40	Bevacizumab + DEX	65 ± 10	15/21	12 months	①②④
					Bevacizumab	64 ± 9	15/19		
Maturi RK 2018 (15)	RCT	USA	Persistent DME	116/129	Ranibizumab+ DEX	64 (59-69)	60/65	6 months	①②④
					Ranibizumab	66(59-71)	56/64		
Ozsaygılı C 2024 (16)	Quasi-RCT	Turkey	DME	82/82	Ranibizumab+ DEX	54.8 ± 6	39/39	12 months	①②④
					Ranibizumab	56.2±2.1	43/43		
Hernández-Bel 2019 (17)	Quasi-RCT	Spain	DME	30/30	Ranibizumab+ DEX	66.2	15/15	13 months	①②
					Ranibizumab	69.4	15/15		
Karimi S 2023 (18)	Quasi-RCT	Iran	DME	81/81	Bevacizumab + DEX	62.66 ± 8.55	40/40	1 month	①②③

DME, diabetic macular edema; DEX - Dexamethasone; RCT, randomized controlled trial; ① BCVA, best-corrected visual acuity; ② CMT,central macular thickness; ③ IOP,intraocular pressure; ④ Adverse reaction.

### Study quality and bias assessment

**Figure 2:**
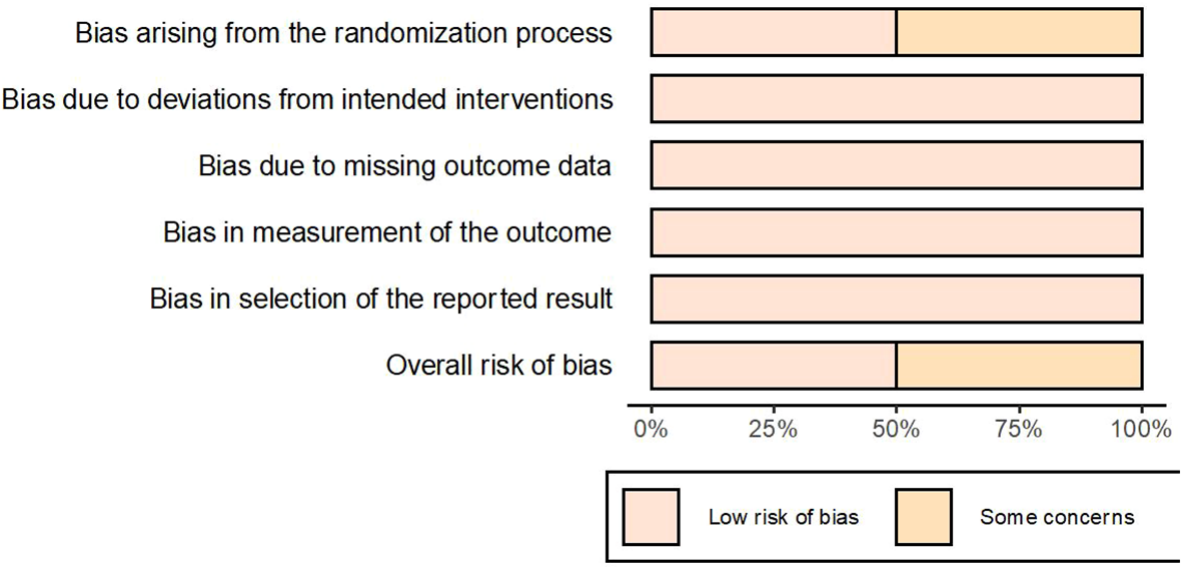
Presents a summary of the risk of bias.

The risk of bias in the included studies was assessed using the Cochrane Risk of Bias 2.0 (ROB 2.0) tool, and the results are summarized in **Table 2**. Among the eight studies, four studies did not provide a clear description of the randomization procedure, and there might be potential risks. Based on the ROB 2.0 criteria, four studies were judged to have some concerns, while the remaining four were rated as low risk. Overall, the methodological quality of the included studies was considered acceptable (**Figure 2**).

**Table 2:** Assessment of the risk of bias in included studies.

Study	D1	D2	D3	D4	D5	Overall
Lin TC 2022 (11)	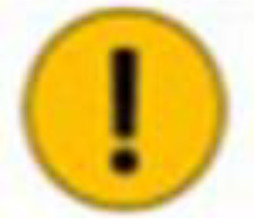					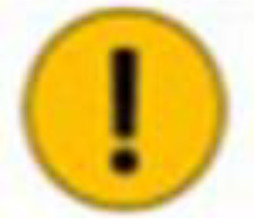
Limon U 2021 (12)						
Kaya M 2021 (13)						
Maturi RK 2015 (14)	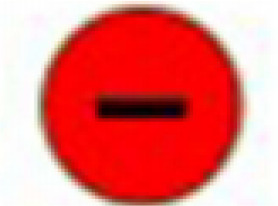					
Maturi RK 2018 (15)						
Ozsaygılı C 2024 (16)	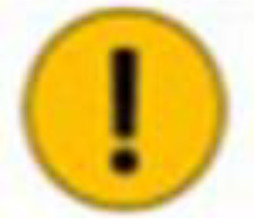					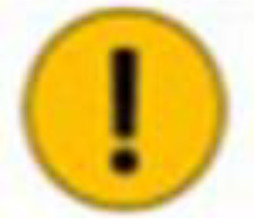
Hernández-Bel 2019 (17)	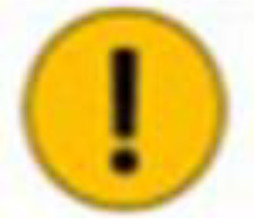					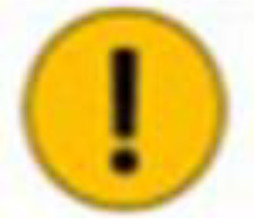
Karimi S 2023 (18)	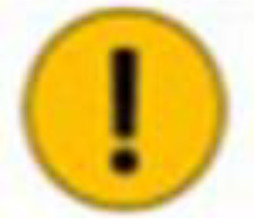					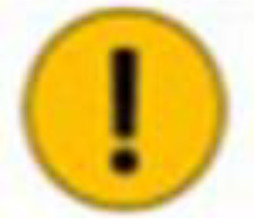

D1, randomization process; D2, deviations from the intended interventions; D3, missing outcome data; D4, measurement of the outcome; D5, selection of the reported result; “!”, some concerns; “+”, low risk; “–”, high risk.

### ETDRS best corrected visual acuity

**Figure 3:**
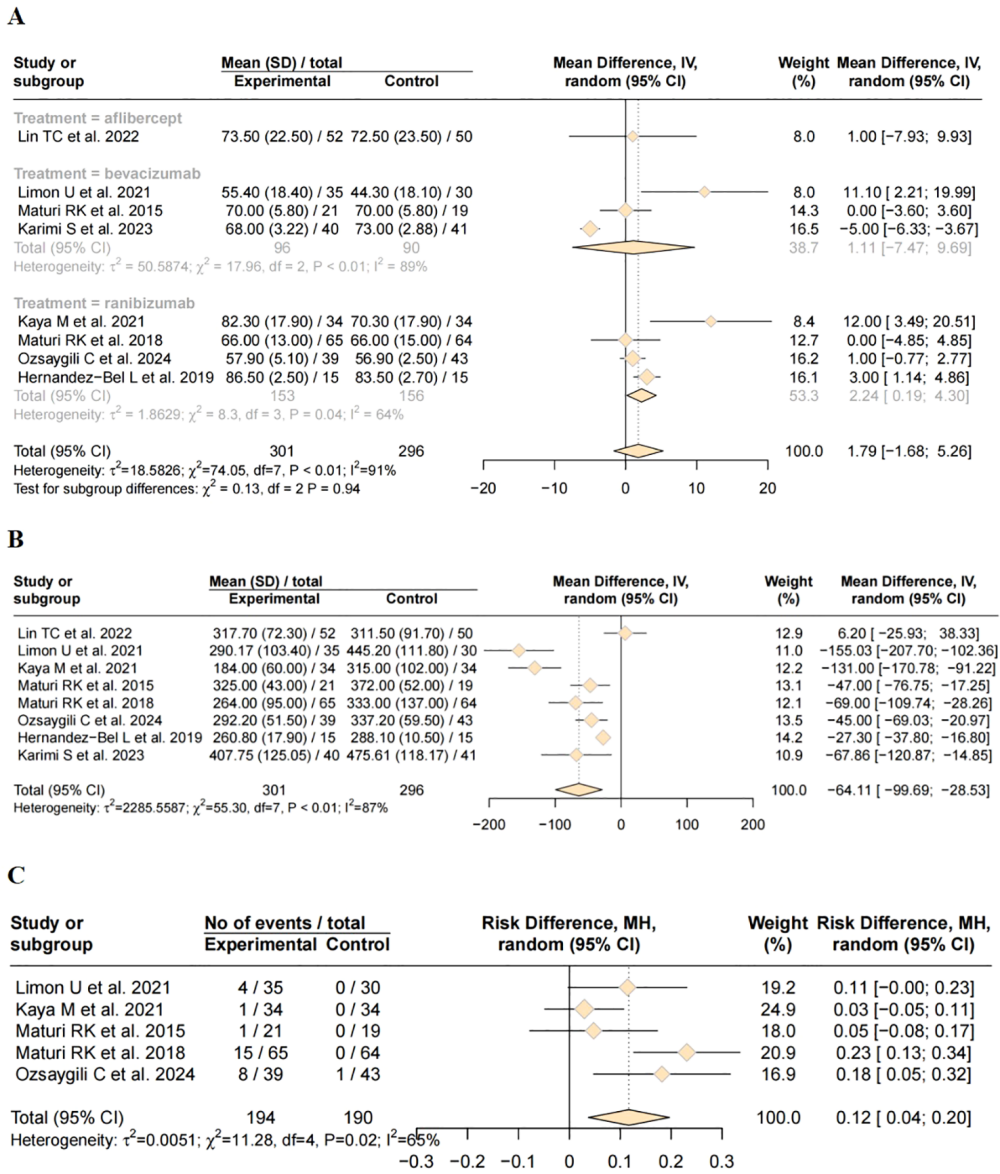
Forest plot of efficacy and safety outcomes after combination therapy of DEX and anti-VEGF versus anti-VEGF monotherapy for DME. **(A)** Best-corrected visual acuity; **(B)** Central macular thickness; **(C)** Incidence of adverse reaction.

All eight studies reported changes in BCVA using ETDRS letter score. A total of 301 patients were included in the combination therapy group and 296 in the monotherapy group. Significant statistical heterogeneity was observed among the studies (I² = 91%, *P* < 0.01). Therefore, a random-effects model was applied for meta-analysis. The pooled results showed no significant difference in BCVA between the combination therapy and monotherapy groups (MD = 1.79, 95% CI: –1.68 to 5.26, *P* = 0.311). To further explore the source of heterogeneity, subgroup analysis was performed based on the type of anti-VEGF agent used, classifying the studies into three subgroups. No significant heterogeneity was observed between subgroups (*P* = 0.94). Only one study was included in aflibercept subgroup. The mean difference in BCVA between the combination and monotherapy groups was 1.11 ETDRS letters (95% CI: –7.47 to 9.69), which was not statistically significant. Substantial heterogeneity was observed (τ² = 50.59, I² = 89%, *P* <no><</no> 0.01), indicating considerable between-study variability, potentially limiting interpretability. Two studies contributed to bevacizumab subgroup. The pooled mean difference favored combination therapy, but the 95% CI again crossed zero (–7.47 to 9.69), suggesting no statistically significant difference. Notably, heterogeneity remained high (I² = 89%), implying methodological or population differences among studies. Notably, in the ranibizumab subgroup, the combination therapy group demonstrated a significantly greater improvement in BCVA compared to the monotherapy group (MD = 2.24, 95% CI: 0.19 to 4.30, *P* <no><</no> 0.05). This suggests a modest but clinically meaningful advantage of the DEX and ranibizumab combination over ranibizumab monotherapy. Detailed results are presented in **Figure 3A**.

### Central macular thickness

All eight included studies reported post-treatment CMT outcomes. A random-effects model was applied due to anticipated clinical and methodological heterogeneity. The overall pooled MD in CMT reduction was -64.11 µm (95% CI: -99.69 to -28.53, *P* < 0.001), favoring the combination therapy group. This statistically significant finding indicates that the addition of DEX results in superior anatomical improvement compared with anti-VEGF therapy alone (**Figure 3B**).

### Intraocular pressure

Three studies reported IOP values following treatment. Due to variability in outcome reporting and measurement units across studies, a quantitative meta-analysis was not feasible, and only descriptive synthesis was performed. One study (10) reported mean post-treatment IOP of 16.3 ± 5.1 mmHg in the combination therapy group and 14.7 ± 3.6 mmHg in the monotherapy group, with no statistically significant difference between groups MD = 1.60, 95% CI: 0.20 to 3.31, *P* = 0.067). Another study (12) observed elevated IOP in 35.3% (12/34) of patients in the combination therapy group, compared to 18.0% (6/34) in the monotherapy group. In contrast, other study (17) reported a reduction in IOP from baseline in both groups: the combination therapy group showed a decrease of 0.65 ± 2.65 mmHg, while the monotherapy group showed a decrease of 0.46 ± 2.05 mmHg. The difference between groups was statistically significant (P = 0.028). Overall, the evidence regarding the effect of treatment on IOP remains inconclusive, and further high-quality studies are needed to clarify the potential IOP-related risks.

### Adverse reaction

Five studies reported the incidence of treatment-related adverse events. Due to variability in outcome definitions and reporting standards across studies, moderate statistical heterogeneity was observed (I² =65%, *P* = 0.02). Therefore, a random-effects model was applied for meta-analysis.The pooled analysis revealed that the difference in adverse event rates between the combination therapy and monotherapy groups was statistically significant (Risk Difference [RD] = 0.12; 95% CI: 0.04 to 0.20, *P* = 0.004), indicating a higher incidence of adverse events in the combination therapy group(**Figure 3C**).

### Subgroup analysis

**Figure 4:**
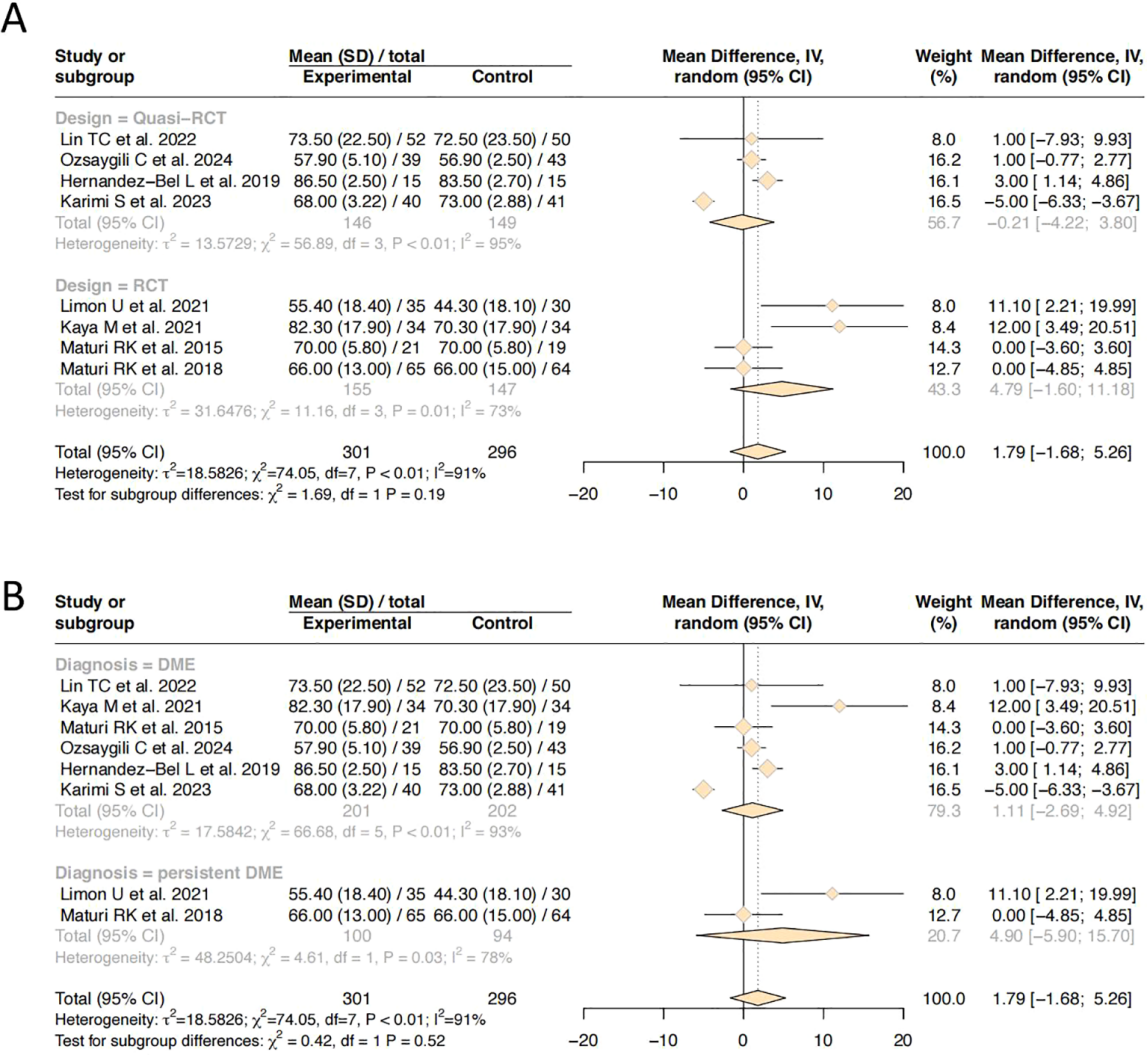
Forest plot of subgroup analysis. **(A)** study design; **(B)** disease diagnosis.

To further investigate sources of heterogeneity in ETDRS score BCVA outcomes, subgroup analyses were performed according to study design (randomized vs. quasi-randomized) and disease diagnosis (general DME vs. persistent DME). No significant heterogeneity was observed between subgroups based on study design (*P* = 0.19), nor among subgroups stratified by disease type (*P* = 0.52) (**Figures 4A, B**). These findings suggest that neither study design nor diagnostic classification contributed meaningfully to between-study heterogeneity, and thus are unlikely to account for the statistical inconsistency observed in the overall analysis.

### Sensitivity analysis and publication bias analysis

**Figure 5:**
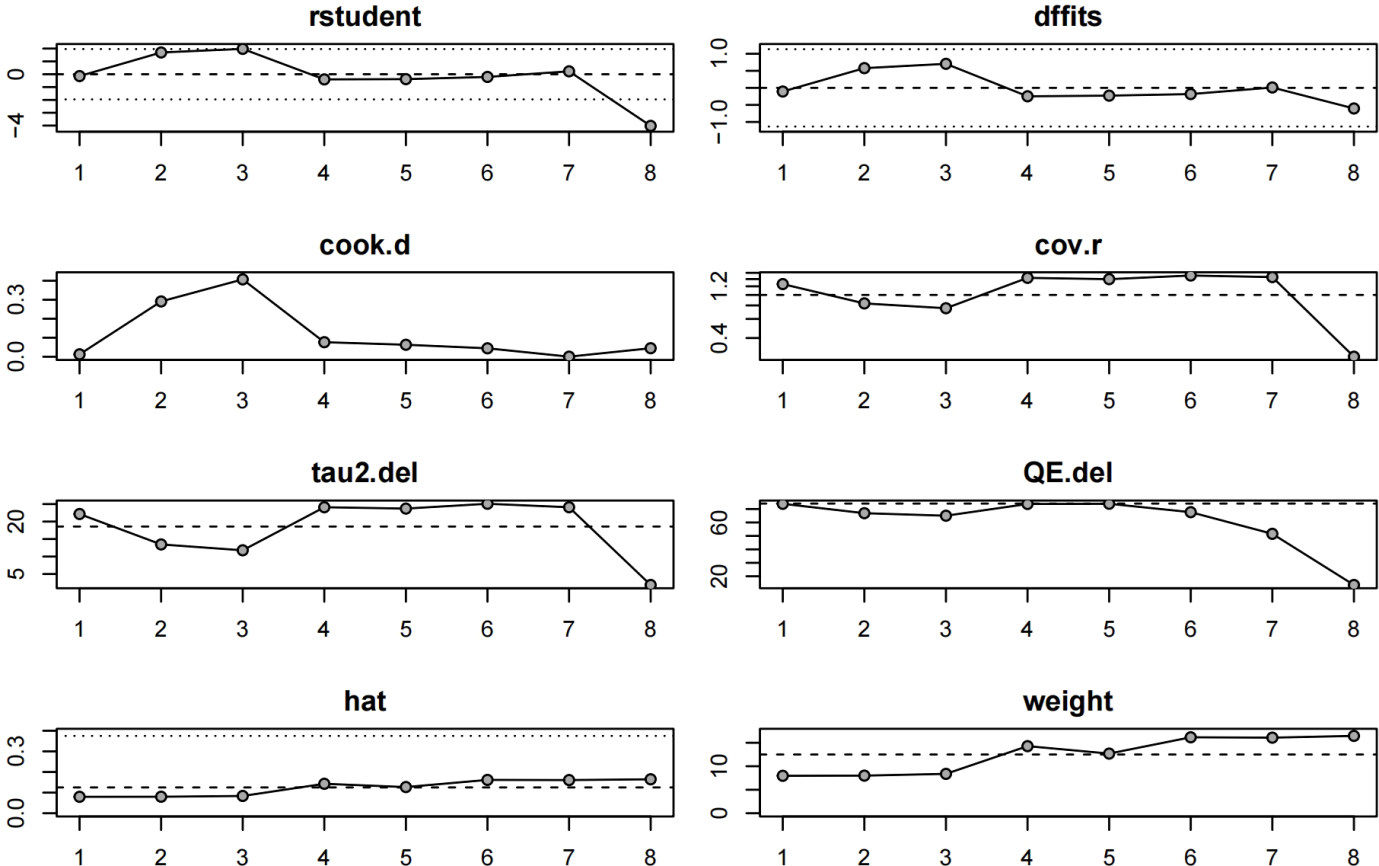
Sensitivity analysis.

**Figure 6:**
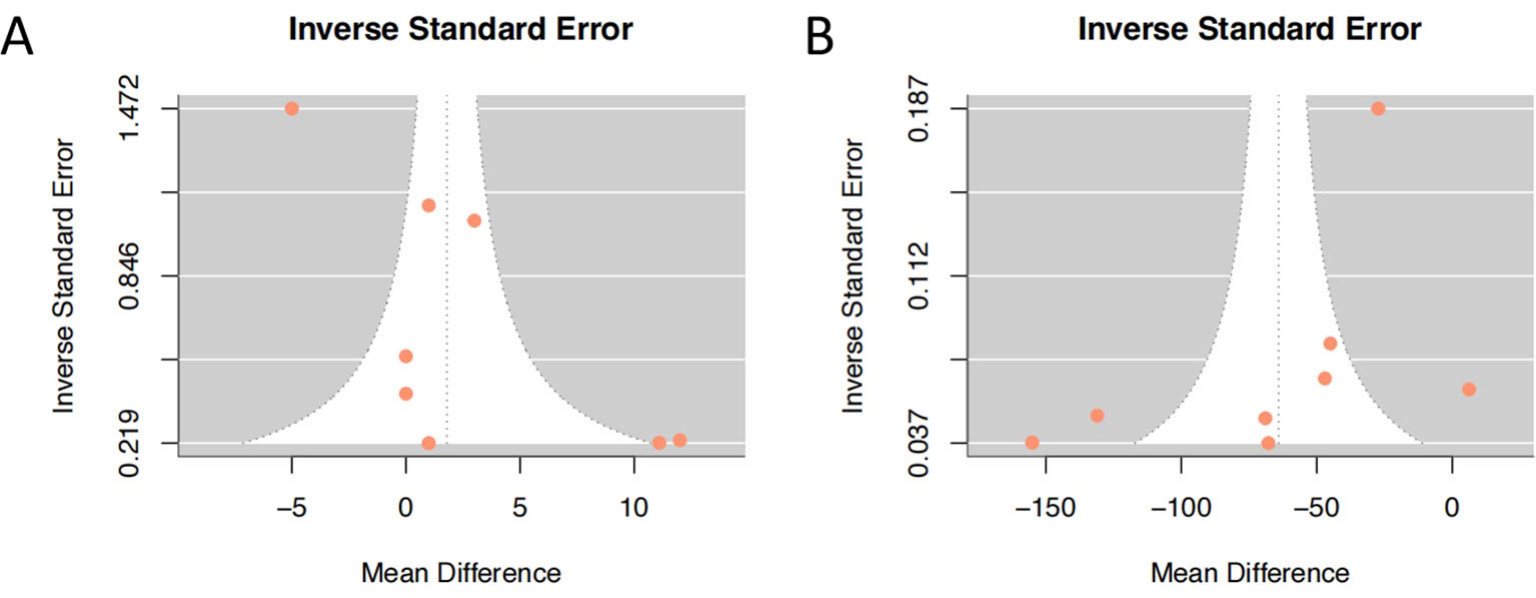
A funnel plot studies assessing the publication bias. **(A)** post-treatment best-corrected visual acuity; **(B)** post-treatment central macular thickness.

We performed an influence diagnostics analysis based on ETDRS score effect estimates to assess the stability of the pooled results. As shown in **Figure 5**, one study (17) was identified as the most influential outlier, likely due to its notably shorter follow-up duration (1 month), compared to other studies which reported outcomes at 6 or 12 months. Nevertheless, exclusion of this study did not substantially alter the overall effect size, suggesting that the main findings are robust and not unduly influenced by any single study. Funnel plots were generated for both post-treatment BCVA and post-treatment CMT outcomes to assess the presence of publication bias (**Figure 6**). The plots showed a generally symmetrical distribution of studies clustered within the funnel boundaries, with no apparent asymmetry, indicating no significant evidence of publication bias.

## Discussion

This meta-analysis provides a comprehensive comparison of the efficacy and safety of DEX implants combined with anti-VEGF drugs versus anti-VEGF agents in the treatment of DME. Our findings indicate that combination therapy involving anti-VEGF agents and intravitreal DEX implants yields a statistically significant reduction in CMT, but does not confer a consistent advantage in BCVA across all studies. The lack of statistically significant improvement in BCVA (MD = 1.79, 95% CI: –1.68 to 5.26, *P* = 0.311) despite robust anatomical gains (MD = –64.11 μm, *P <* 0.001) aligns with earlier clinical observations suggesting a dissociation between structural and functional recovery in DME, particularly in cases with chronic or irreversible photoreceptor damage (19). Interestingly, subgroup analysis revealed a significant BCVA improvement in the ranibizumab-DEX subgroup (MD = 2.24, *P* < 0.05), potentially reflecting pharmacodynamic synergy or superior pharmacokinetic compatibility between these agents. However, the high heterogeneity in visual outcomes (I² = 91%) suggests that differences in follow-up duration, baseline disease severity, or prior treatment status may substantially influence therapeutic response. In our subgroup analysis, the combination of DEX with ranibizumab resulted in a statistically significant improvement in BCVA compared to ranibizumab monotherapy, whereas the addition of DEX to either bevacizumab or aflibercept did not yield similar functional benefits. Nonetheless, this observation should be interpreted with caution. The limited number of included studies and the small sample sizes within subgroups restrict the generalizability of these findings. Therefore, further large-scale, head-to-head trials are warranted to investigate the potential synergistic effects of combining DEX with different anti-VEGF agents across varying DME phenotypes.

Although our meta-analysis did not demonstrate a statistically significant superiority in BCVA with combined DEX and anti-VEGF therapy versus anti-VEGF monotherapy, it is noteworthy that BCVA consistently improved from baseline in all combination-treated groups across the included studies. This suggests that, while the visual gains may not significantly exceed those achieved by anti-VEGF alone, the combination therapy still provides clinically meaningful benefit. DEX robustly induces lipocortins, especially annexin-A1, which serve as potent endogenous anti-inflammatory mediators (20). Annexin-A1 inhibits phospholipase A_2_activity, thereby reducing leukocyte chemotaxis, and suppressing the biosynthesis of prostaglandins and leukotrienes through preventing arachidonic acid release from membrane phospholipids, key drivers of intraocular inflammation (21, 22). Through these mechanisms, DEX effectively stabilizes the blood-retina barrier and mitigates vascular leakage and macular edema, contributing to improved visual outcomes (23).

Subgroup analysis based on the specific anti-VEGF agents used offered additional insights. Notably, in the ranibizumab subgroup, combination therapy with DEX was associated with a statistically significant improvement in ETDRS letter scores compared to monotherapy. This suggests that ranibizumab may exert additive or synergistic effects when used alongside corticosteroids, possibly due to complementary mechanisms targeting both angiogenic and inflammatory pathways.The absence of a significant difference in the overall pooled result, despite some positive trends in individual studies, may also reflect differences in retreatment criteria, injection frequency, and definitions of treatment resistance. In addition, ceiling effects may have occurred in patients with relatively preserved baseline visual acuity, limiting the potential for measurable improvement. In patients with resistant DME, defined as those with poor responses to multiple prior anti-VEGF injections, DEX implants significantly outperformed anti-VEGF monotherapy, confirming the therapeutic potential of corticosteroids in this challenging subgroup.

DEX is widely regarded as one of the most potent corticosteroids in ophthalmology, exerting its multifaceted therapeutic effects through the modulation of diverse signal transduction pathways. Regarding anatomical improvement, our findings indicate that DEX implants consistently achieved greater reductions in CMT at both short- and medium-term follow-ups compared to anti-VEGF therapy. This suggests that corticosteroids’ anti-inflammatory mechanism may more effectively address the vascular permeability and inflammatory components of DME pathogenesis, particularly in cases with severe baseline edema.These results are in line with other study that highlight the superior short-term anatomic efficacy of DEX implants, particularly in CMT reduction (24, 25). The enhanced efficacy of DEX in managing persistent or refractory DME can likely be attributed to its robust anti-inflammatory, anti-edematous, and anti-angiogenic properties. As previously established, corticosteroids downregulate the expression of VEGF and multiple proinflammatory cytokines, reduce vascular leakage, and inhibit leukocyte adhesion and leukostasis (26). However, the substantial heterogeneity observed across the included studies cautions against broad generalizations of these findings. Subgroup and sensitivity analyses are essential to elucidate the specific patient subgroups that derive the greatest benefit from combination therapy. Furthermore, it is critical to recognize that anatomical improvements, such as reductions in CMT, do not consistently translate into enhanced visual acuity outcomes. Indeed, prior investigations have demonstrated a weak or inconsistent correlation between structural metrics and functional vision gains, underscoring the need to concurrently assess both parameters in future studies of diabetic macular edema treatment (27).

Our pooled analysis identified a significantly higher incidence of adverse events in the combination therapy group, which aligns with the known corticosteroid-related complications. Consistent with prior evidence, our meta-analysis confirms that intravitreal DEX implants are associated with ocular adverse events, most notably IOP elevation and cataract progression. IOP elevation was observed as early as three months following DEX injection and persisted in a subset of patients, underscoring the need for long-term IOP monitoring. Cataract formation, particularly in phakic eyes, was another notable complication, emphasizing the importance of careful patient selection. In contrast, anti-VEGF agents demonstrated a more favorable ocular safety profile, with significantly lower rates of ocular hypertension and lens-related complications. While DEX implants provide substantial anatomical and short-term visual benefits, their risk profile warrants individualized treatment strategies, especially in younger or phakic patients who may be more vulnerable to steroid-induced adverse events. Although combination therapy with DEX and anti-VEGF agents appears to improve anatomical outcomes, its safety remains a critical consideration.

Several limitations of this meta-analysis warrant careful consideration. First, moderate to high heterogeneity was observed across most primary outcomes, a phenomenon often rooted in differences in study design, treatment regimens, DME subtypes, dosing protocols, and follow-up durations. Such variability reduces the generalizability of pooled estimates. Second, the conversion of BCVA from logMAR to ETDRS letters, often necessitated by inconsistent reporting formats, introduces potential variability, particularly when original data derive from non-standard visual acuity charts. Third, the time points for outcome assessments were inconsistently reported across included studies, limiting our ability to stratify results by specific follow-up intervals. Future meta-analyses could address this limitation by implementing standardized temporal subgroup analyses. Future meta-analyses should prioritize standardized outcome time points to enable robust, interval-specific comparisons.

Looking forward, well-designed, large-scale randomized controlled trials with harmonized treatment protocols, clearly defined outcome measures, and extended follow-up are essential to validate our findings. Furthermore, the integration of biomarker-driven and imaging-guided approaches, such as optical coherence tomography, derived parameters or inflammatory cytokine profiling may facilitate more personalized treatment strategies. Finally, the inclusion of patient-centered outcomes such as treatment burden, health-related quality of life, and cost-effectiveness will be critical for informing real-world clinical decision-making and health policy.

The rationale for combining anti-VEGF agents with intravitreal DEX lies in their mechanistically distinct yet synergistic modes of action: anti-VEGF therapies mitigate vascular hyper-permeability by directly antagonizing VEGF signaling, whereas corticosteroids suppress a broad range of pro-inflammatory cytokines and enhance blood-retinal barrier integrity. These complementary effects offer a pathophysiologically rational strategy for managing DME, particularly in patients with a suboptimal response to anti-VEGF monotherapy or in pseudophakic individuals, where corticosteroid-related cataractogenesis is less concerning (28). The observed anatomical improvements and potential reduction in treatment burden further support the clinical utility of this combination approach. Nonetheless, the lack of consistent long-term functional gains and a heightened risk of corticosteroid-associated adverse events underscore the need for prudent patient selection and vigilant follow-up. Future well-designed randomized controlled trials with extended follow-up, standardized endpoints, and stratification based on inflammatory biomarkers or DME chronicity are warranted to optimize patient-specific therapeutic algorithms and clarify the long-term role of combination regimens in DME management.

## Conclusions

This meta-analysis demonstrates that combination therapy with intravitreal DEX and anti-VEGF agents offers superior anatomical efficacy, particularly in reducing CMT, compared to anti-VEGF monotherapy in the treatment of DME. While combination therapy was associated with an increased incidence of ocular adverse events, notably IOP elevation and ocular hypertension, these events were generally mild and clinically manageable with appropriate monitoring and intervention. Importantly, our findings did not reveal a statistically significant improvement in BCVA with combination therapy compared to anti-VEGF monotherapy in the overall DME population. However, the ranibizumab subgroup analyses suggested that patients with persistent or refractory DME may derive greater visual benefit from the addition of DEX, though this observation requires confirmation through larger, high-quality randomized controlled trials.

In summary, DEX combined with anti-VEGF agents represents a promising treatment strategy for selected DME patients, offering enhanced anatomical outcomes and the potential to reduce treatment burden. Nevertheless, the elevated risk of steroid-related ocular complications underscores the importance of individualized treatment decisions and close ophthalmic monitoring. Future studies should focus on refining patient selection criteria and standardizing treatment protocols to optimize clinical outcomes.
